# Supplementary material for: Systematic identification of disease-causing promoter and untranslated region variants in 8,040 undiagnosed individuals with rare disease
Source: medRxiv. 2023 Sep 12:2023.09.12.23295416. Preprint. [Version 1] doi: 10.1101/2023.09.12.23295416 (PMC10516070; doi:10.1101/2023.09.12.23295416)
Supplement: Supplement 1 [file media-1.pdf]

Supplementary section

Supplementary Tables

Supplementary Table 1: Total, and mean size of near-coding region annotations by type.

Region	Total (bp)	Mean per gene (bp)
Promoter	543,422	347.9
5’UTR	372,417	239.2
5’UTR intron	15,795,406	10080.0
3’UTR	3,437,362	2194.7
3’UTR intron	263,304	168.0
All Regions	20,409,032	13029.8

Supplementary Table 2: Genomic coordinates of analysed near-coding regions across green dominant PanelApp genes.

Hosted on GitHub due to size:

https://github.com/Computational-Rare-Disease-Genomics-WHG/Near_coding_annotation

Supplementary Table 3: Total and mean variants across promoters and full MANE transcript length, in case and control participants in matched pairs, by genetically inferred ancestry group, and sex (AF 0.0001).

Sex	Genetically inferred ancestry	Case participants	Control participants	Mean Case variants	Mean Control variants
Female	European	7,827	5,198	430.38	428.04
Male	European	8,096	4,881	430.47	427.91
Female	South Asian	729	538	619.10	629.93
Male	South Asian	989	610	629.94	631.29

Supplementary Table 4: Classifications for the six newly identified DNVs according to the ACMG/AMP guidelines and adaptations for variants in non-coding regions(8,61). Thresholds for PP3 were taken from Pejaver *et al*. 2022 (doi: 10.1016/j.ajhg.2022.10.013) for PhyloP and CADD, and Walker *et al*. 2023 (doi: 10.1101/2023.02.24.23286431) for SpliceAI. *Functional data from Willemsen *et al*. 2017 (doi: 10.1038/ejhg.2017.45).

Variant	Gene	Applied rule codes	Classification
chr1:g.244051270 C>T	*ZBTB18*	PS2 (de novo); PP3_supporting (PhyloP score); PM2_supporting (absent from gnomADv3.1.2)	Likely Pathogenic

chr1:g.42958758 C>T	*SLC2A1*	PS2 (de novo); PP3_supporting (UTRannotator prediction); PM2_supporting (absent from gnomADv3.1.2); PS3_supporting (published functional studies*)	Likely Pathogenic
chr3:g.9397978 G>A	*SETD5*	PS2 (de novo); PP3_moderate (SpliceAI score); PM2_supporting (absent from gnomADv3.1.2); PP4_supporting (methylation studies confirm *SETD5*); PS1_supporting (same nucleotide)	Likely Pathogenic
chr3:g.9397974 CAAGGT>C	*SETD5*	PS2 (de novo); PP3_moderate (SpliceAI score); PM2_supporting (absent from gnomADv3.1.2); PP4_supporting (methylation studies confirm *SETD5*); PS1_supporting (same nucleotide)	Likely Pathogenic
chr5:g.36953601 T>A	*NIBPL*	PS2 (de novo); PP3_moderate (SpliceAI score); PM2_supporting (absent from gnomADv3.1.2)	Likely Pathogenic
chr20:g.58909654 A>G	*GNAS*	PS2 (de novo); PM2_supporting (absent from gnomADv3.1.2); PS3_supporting (evidence of aberant splicing and significant reduction in expression compared to 499 controls shown in RNA-seq)	Likely Pathogenic

Supplementary Table 5: Burden testing of prioritised variants in case and control participants by region and variant annotation. The proportion of total participants (N=7,862) are shown in the control, and case

participant columns in round brackets, and 95% confidence intervals are shown in square brackets alongside the odds ratios.

Region	Control Participants	Case Participants	Fisher's *P*-value	Odds Ratio
ALL	775 (9.86%)	837 (10.65%)	0.109	1.090 [0.981,1.210]
Promoter	107 (1.36%)	105 (1.34%)	0.945	0.981 [0.741,1.299]
5'UTR	164 (2.09%)	182 (2.31%)	0.356	1.112 [0.894,1.386]
5'UTR intron	103 (1.31%)	137 (1.74%)	0.032	1.336 [1.025,1.746]
3'UTR	471 (5.99%)	459 (5.84%)	0.710	0.973 [0.850,1.113]
3'UTR intron	3 (0.04%)	11 (0.14%)	0.057	3.670 [0.969,20.494]
**Annotation**				
CADD	38 (0.48%)	57 (0.73%)	0.063	1.504 [0.979,2.333]
PhyloP	462 (5.88%)	473 (6.02%)	0.736	1.025 [0.896,1.173]
TFBS	53 (0.67%)	51 (0.65%)	0.922	0.962 [0.641,1.442]
KOZAK	3 (0.04%)	7 (0.09%)	0.344	2.335 [0.533,13.995]
IRES	0 (0.00%)	1 (0.01%)		
UTR Annotator	82 (1.04%)	94 (1.20%)	0.404	1.148 [0.843,1.566]
SpliceAI	149 (1.90%)	199 (2.53%)	0.008	1.344 [1.079,1.678]
PolyA	35 (0.45%)	41 (0.52%)	0.566	1.172 [0.728,1.898]
RBP	25 (0.32%)	35 (0.45%)	0.244	1.402 [0.815,2.446]

dORF	7 (0.09%)	2 (0.03%)		

Supplementary Figures

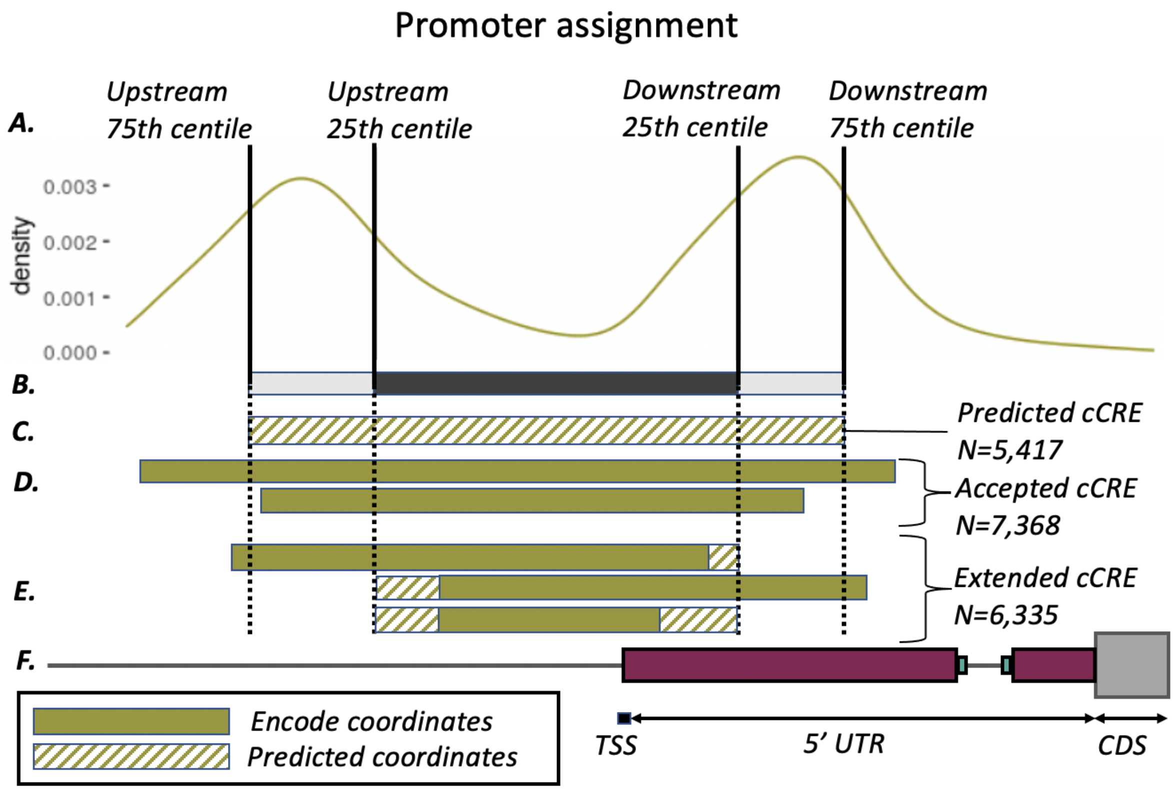

Supplementary Figure 1: Graphical representation of promoter assignment with reference to the TSS (F). For all cCREs that overlapped a TSS the total distance the cCRE extends in each direction (upstream/5' and downstream/3') was calculated (A). The 25th and 75th percentiles of the distance in each direction was calculated to form the basis of a 'minimal' (B, dark grey) and 'maximal' (B, light grey) promoter. For genes without an overlapping cCRE a promoter was predicted using the 'maximal' promoter criteria (C, shaded line). For genes with an overlapping cCRE that extended beyond the 25th centile in both directions (D) the cCRE was accepted without modification. For genes where the overlapping cCRE

(E, solid line) fell short of the 25th centile in either direction, this was extended to match the coordinates of the ‘minimal’ promoter (E, shaded line).

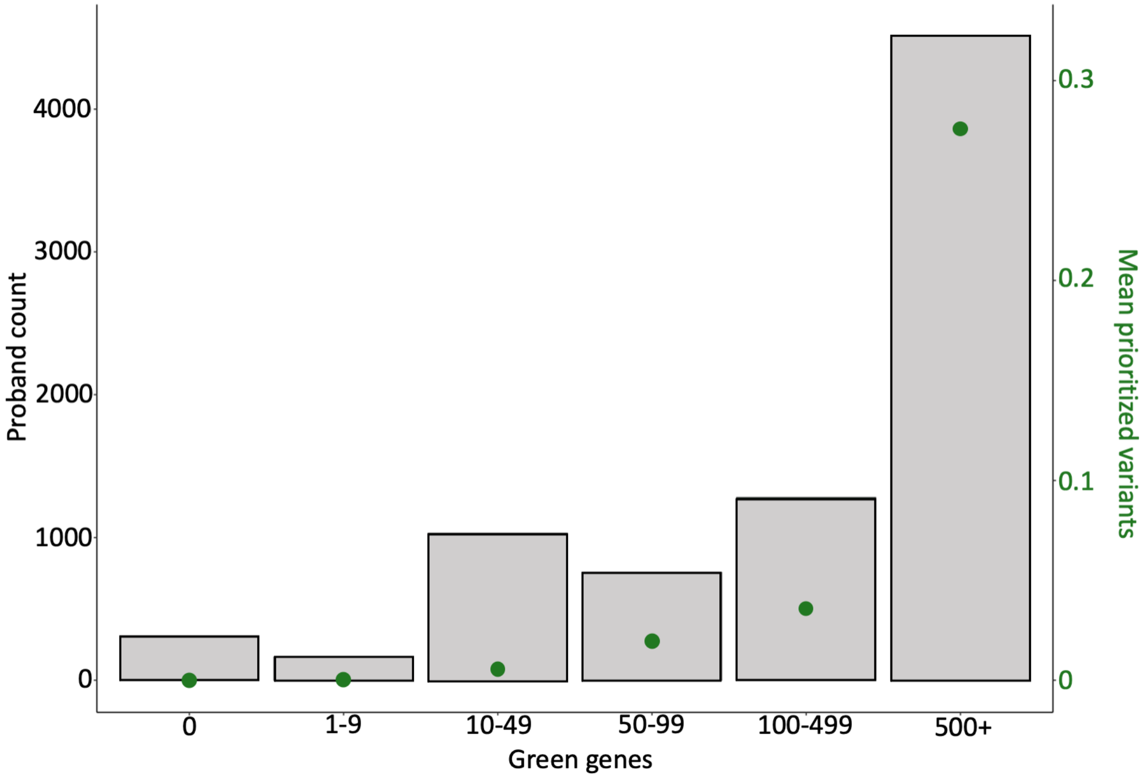

Supplementary Figure 2: The number of probands with different numbers of assigned green-dominant genes (grey bars) and mean prioritised variants from the *de novo* variant dataset (green dots) in 8,040 probands.

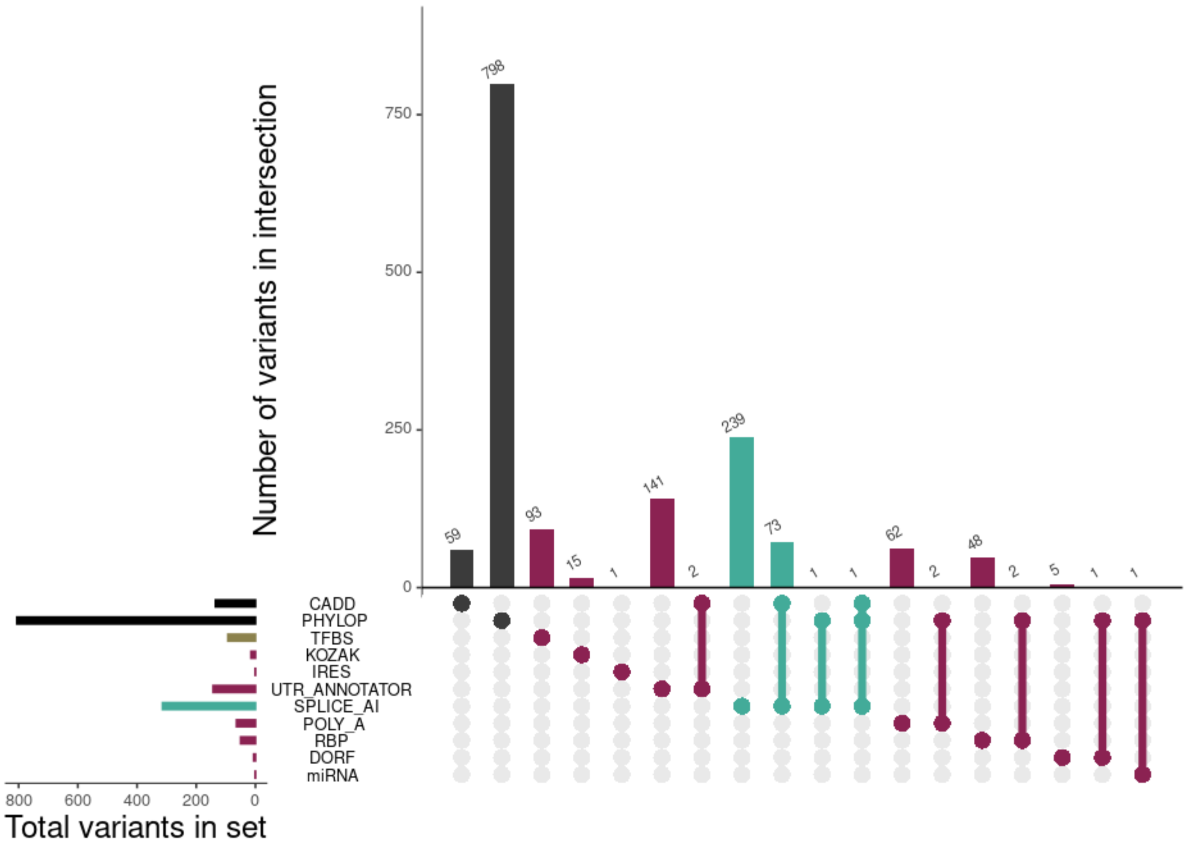

Supplementary figure 3: Annotated variants in the aggregated variant set used for burden testing. Universal variant annotations are shown in black, UTR specific variant annotations in raspberry, promoter specific variant annotations in mustard, and intronic / splicing variant annotations in teal. Vertical bars in the top panel denote the number of variants identified with specific region and variant annotations that are represented by the bar colour (regions), and in the upset plot below (variant annotations). The total number of DNVs with each variant annotation is shown by the horizontal bars to the left of the upset.

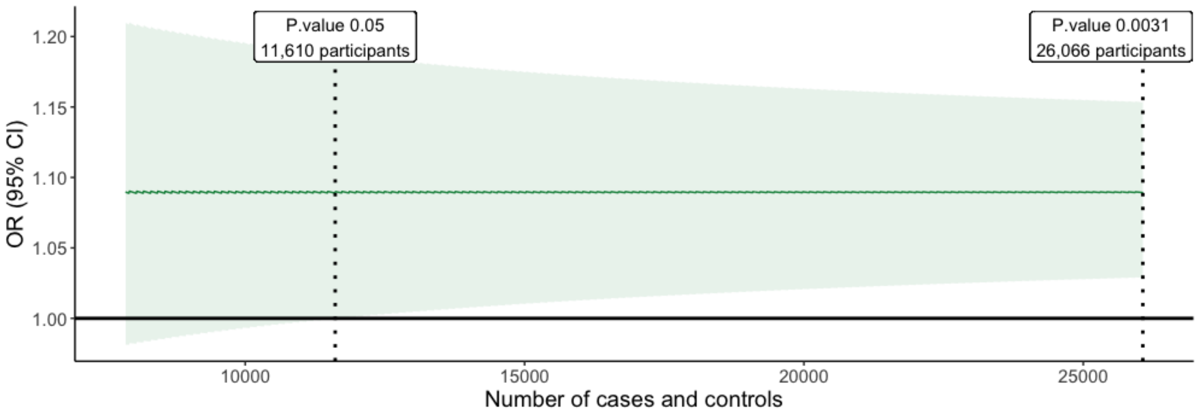

Supplementary figure 4: Estimated number of participants required to reach significance of *P<0.05* (left dotted line) and *P<0.0031* (right dotted line; Bonferroni adjusted threshold accounting for 16 tests) across all region and variant annotations. Odds ratio (OR) is shown in dark green, with 95% confidence interval shaded in light green. OR=1 is marked with a black solid line.
